# Morphofunctional State and Circadian Rhythms of the Liver of Female Rats under the Influence of Chronic Alcohol Intoxication and Constant Lighting

**DOI:** 10.3390/ijms231810744

**Published:** 2022-09-15

**Authors:** David A. Areshidze, Maria A. Kozlova

**Affiliations:** Avtsyn Research Institute of Human Morphology of Federal State Budgetary Scientific Institution “Petrovsky National Research Center of Surgery”, 117418 Moscow, Russia

**Keywords:** hepatocyte, female, liver, alcohol, constant lighting

## Abstract

A separate and combined effect of constant illumination and chronic alcohol intoxication (CAI) on diurnal dynamics of micromorphometric parameters of hepatocytes in female Wistar rats and *p53*, *Ki-67*, *PER2*, *BMAL1,* and *ADH5* expression in these cells were studied. The increase in apoptotic activity and proliferation in all animals under the action of chronodestructors is shown. All experimental animals showed a decrease in *BMAL1* expression and increase in *PER2* expression; *ADH5* is overexpressed under the influence of ethanol. Circadian rhythms (CRs) of *BMAL1*, *PER2*, *p53*, and *Ki-67* expression persist in all groups, except combined action of chronodestructors, and *ADH5* CRs persist in all groups—thus, these rhythms in females are quite stable. CRs of the hepatocyte nuclei area are preserved in all the studied groups, although they undergo a significant shift. At the same time, the CRs of the hepatocyte area are destroyed under the action of light, both independently and in combination with CAI, and the CR of the nuclear-cytoplasmic ratio (NCR) is destroyed by exposure to CAI. It can be assumed that CRs of the hepatocyte area are significantly affected by dark deprivation and NCR rhythm is sensitive to ethanol consumption, while the stability of studied genes’ expression rhythms at separate influences of studied chronodestructors is maintained by yet unknown adaptation mechanisms. It is necessary to note that, according to our previous studies of male rats, rat females show significantly greater stability of the studied CRs.

## 1. Introduction

Circadian rhythms (CRs) are biological processes that proceed with a period of about 24 h and provide synchronization of biological processes with environmental factors, which is crucial for maintaining homeostasis, as well as adapting to changing environmental conditions [1]. They are associated with the light–dark cycle, causing the existence of cycles of sleep–wake, feeding–starvation, secretion of hormones, the exchange of energy resources, and body temperature [2]. CRs are described in living organisms ranging from bacteria to mammals [3].

Being genetically determined, the temporal organization of systems of organism, nevertheless, is quite plastically modulated by the influence of periodic environmental factors [4,5]. The CRs of separate physiological processes of various systems are strictly synchronized with each other. This synchronization determines the necessary order and optimal coherence of these processes between themselves and the external environment [6,7]. The degree of synchronization of the mammalian CRs is a reflection of the degree of its adaptation to existence in specific environmental conditions. In cases of successful adaptation processes, the grade of impact of stressors on circadian rhythms is insignificant. Otherwise, the rhythmic processes of the organism lose their correctness, regularity, and chronodisruption occurs, which can lead to the development of diseases and pathological conditions [8,9].

In hepatocytes, as well as in cells of other organs, the biological clock at the molecular level is generated by a core transcription and translation feedback loop comprising the clock genes *BMAL1 (Brain and muscle ARNT-Like 1)*, *CLOCK (Circadian Locomotor Output Cycles Kaput)*, *PER1* (period 1) and *PER2 (period 2)*, and *CRY1* (cryptochrome 1) and *CRY2 (cryptochrome 2)*. BMAL1 and CLOCK proteins form a heterodimer that activates transcription of *PER* and *CRY* genes and other clock-controlled genes by binding to E-box response elements within their promoters. CRY and PER proteins are subsequently phosphorylated by CKI (casein kinase I) ε or CKIδ (casein kinase I delta) and translocate into the nucleus, where they repress the transcriptional activity of BMAL1 and CLOCK proteins. PP1 (protein phosphatase 1) dephosphorylates PER proteins, shortening the oscillation period of clock genes. Thus, the balance of CKI and PP1 activity determines the circadian oscillator period. In addition, other feedback loops are interwoven into the core clock feedback loop. Transcription of *BMAL1, CRY1, NPAS2* (neuronal PAS domain-containing protein 2, a paralogue of *CLOCK*), and *NFIL3* (nuclear factor, interleukin 3 regulated, also known as E4BP4, a negative regulator of *PER1* and *PER2*) is repressed by REV-ERBα (also known as nuclear receptor subfamily 1 group D member 1, NR1D1) and REV-ERBβ (nuclear receptor subfamily 1 group D member 2, NR1D2) and activated by ROR (RAR-related orphan receptor) α, RORβ, and RORγ via binding of their ROR-responsive element promoter regions. The Rev-erb and Ror families of genes are in turn positively regulated by CLOCK and BMAL1 proteins [10,11].

Most of liver functions follow a circadian rhythmicity [12,13]. Expression of genes, which provide the wide spectrum of liver functions, can be regulated as directly by the autonomic circadian system of hepatocytes, by rhythmic signals from the external environment (light, food intake), or by a combination of both mechanisms [14,15]. At the same time, a high degree of dependence of the functioning of the liver on the normally synchronized control of its CRs by the suprachiasmatic nuclei of hypothalamus (SCN) and the pineal gland was proved [16,17]. There is an opinion that light, not being directly a factor critical for maintaining circadian rhythms, affects their period and amplitude, the expression of some genes, and the coordination of rhythms among themselves [18].

Violation of the normal circadian rhythmicity of the functioning of the liver is considered as one of the leading factors in the development of non-alcoholic fatty liver disease (NAFLD), non-alcoholic steatohepatitis (NASH), and a number of other diseases [19,20]. Such disturbances of the normal functioning of clock genes in hepatocytes are associated with a number of liver dysfunctions and pathologies [21,22]. In particular, the disruption of normal functioning of *PER* gene family at hepatitis was established [23].

The relationship between disorders of the liver CRs and the risks of developing oncological diseases is also known [24,25]. Abnormal expression of the main clock genes was found in human hepatocellular carcinoma (HCC) biopsy tissue samples. Chronic jetlag also induces spontaneous HCC in wild-type mice. The process begins with non-alcoholic fatty liver disease that progress to NASH, cirrhosis, and HCC. This path is based on gene dysregulation caused by jetlag, dysfunction, and circadian desynchronization of metabolic processes in the liver [26].

The role of clock genes in the regulation and circadian rhythm of apoptosis is described [27]. In addition to clock genes, melatonin is involved in the regulation of CRs of apoptosis [28,29].

Constant lighting is one of the leading causes of pronounced morphofunctional changes in the mammalian organs and systems of organs. Due to social reasons, there is an extensive number of sources of artificial lighting at night in the modern world, whose influence leads to a shift in the circadian rhythms of the whole organism of humans and other species and the development of desynchronization in it [30]. One of the causes of such desynchronization is shift work, which is now considered the main factor in violation of the structure and coordination of the CRs of an organism of humans [31]. Change in rhythmicity in peripheral organs under conditions of constant lighting may be linked with the disorder in CRs of SCN [32], production of melatonin by the pineal gland, or changes that occur directly in the tissues [33,34]. A significant amount of data indicating changes in the CRs of peripheral organs of mammals under conditions of dark deprivation have been collected to date [35,36,37].

Another significant factor that causes chronodisruption in humans is alcohol. It is shown that that circadian desynchronization is one of the signs of alcoholism [38,39,40]. There are interesting links between alcohol consumption and circadian rhythm disturbance: just as alcohol alters the functioning of the SCN, disruption of the normal circadian rhythm causes alcohol cravings [41,42,43]. In addition, alcohol and its metabolites have an impact both on the functioning of peripheral pacemakers and directly on the work of clock genes [44,45]. Circadian rhythm disorders of various etiologies are important, if not decisive, in increasing the susceptibility of the liver to alcohol-induced damage and play a defining role in the severity of alcohol pathology [46,47]. The significant way in which ethanol influences the structure of CRs of an organism is its pineal-mediated effect. The use of even small doses of alcohol for 14 days causes a phase shift in the circadian rhythm of melatonin [48], and in alcoholics, the production of nocturnal melatonin is significantly reduced [49,50].

The question of sex differences in the circadian rhythmicity of the mammalian organism is very complicated. Just a few publications are devoted to the CRs of the liver, including the influence of its rhythmic work on processes in other tissues. Traditionally, male experimental animals are used in most studies of mammalian circadian rhythms [51,52], and only 20% of studies are devoted to the study of females [53].

A number of liver functions has differences depending on sex. It is primarily the sex-specific production of liver proteins, xenobiotic transporters, and cytochrome *P450* enzymes involved in the metabolism of sex steroids [54]. According to the current point of view, the liver of females is characterized by a highly efficient metabolic phenotype, increased biogenesis, which is necessary for a successful course of pregnancy [55].

Our previous studies showed that in male rats, the constant illumination and CAI, both with separate and combined action of these factors, led to the destruction of the CRs of expression of *Ki-67 (marker of proliferation Ki-67)*, *PER2*, *p53 (tumor protein p53 gene)*, *BMAL1*, *ADH5 (alcohol dehydrogenase 5 (Class III) gene)*, and also of a number of biochemical parameters and micromorphometric characteristics of hepatocytes with background of pathological structural changes in the liver [56,57,58].

It is considered that the sexual dimorphism of the liver depends on the pulsating (in males) and constant (in females) secretion of growth hormones, as well as on the influence of androgens and estrogens [59,60,61].

Studies on the daily dynamics of lipid and carbohydrate metabolism allowed the establishment of the presence of their CRs in mammals of both sexes, but there were gender differences in the mesor, acrophase, and amplitude of rhythms [62]. Sexual dimorphism of circadian rhythms is also established for nuclear peroxisome proliferator-activated receptors (PPAR) playing an important role in the initiation of the inflammatory response [63]. Sexual dimorphism is also described for the daily dynamics of lipid metabolism and *BMAL1* expression and for the expression of genes that ensure the functioning of the antioxidant system of the liver [64,65].

Anyway, the issue of sexual dimorphism of liver circadian rhythms remains poorly understood now. We conducted the study on the effect of constant lighting, chronic alcohol intoxication, and the combined action of these factors on the morphometric parameters of hepatocytes (the linear dimensions of hepatocytes and their nuclei, nuclear-cytoplasmic ratio). A number of other micromorphometric parameters are significant indicators for assessing the state and functional changes in the liver [65,66,67], on levels of expression of *Ki-67*, *PER2*, *BMAL1*, *p53,* and *ADH5* in hepatocytes, on daily dynamics of these parameters, and also on morphological conditions of the liver in female Wistar rats.

## 2. Results

### 2.1. Influence of Constant Lighting and CAI on the Morphofunctional Condition of the Liver

**Figure 1:**
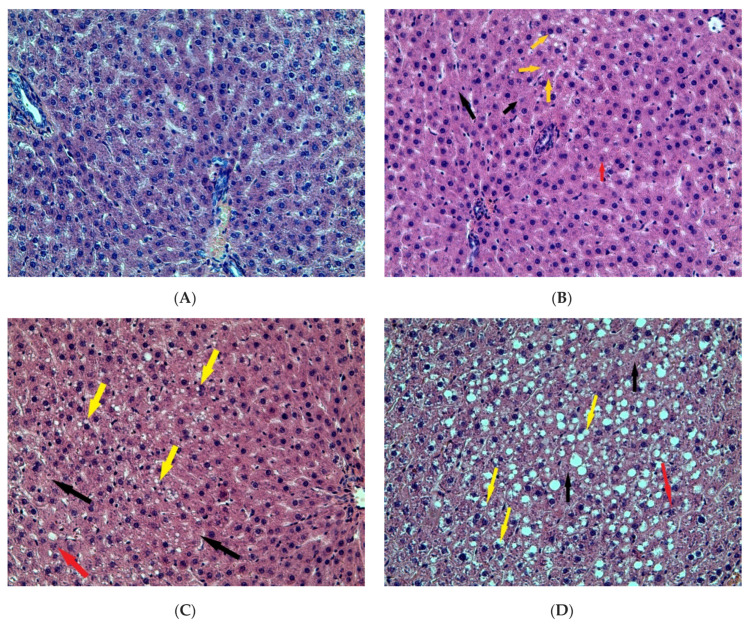
Liver of rats: (**A**)—control; (**B**)—EtOH; (**C**)—CL; (**D**)—EtOH + CL. Hematoxylin and eosin staining, ×200. Yellow arrows indicate hepatocytes in a state of fatty degeneration; red arrows—hepatocytes in a state of apoptosis; black arrows—hepatocytes in a state of necrosis.

**Figure 2:**
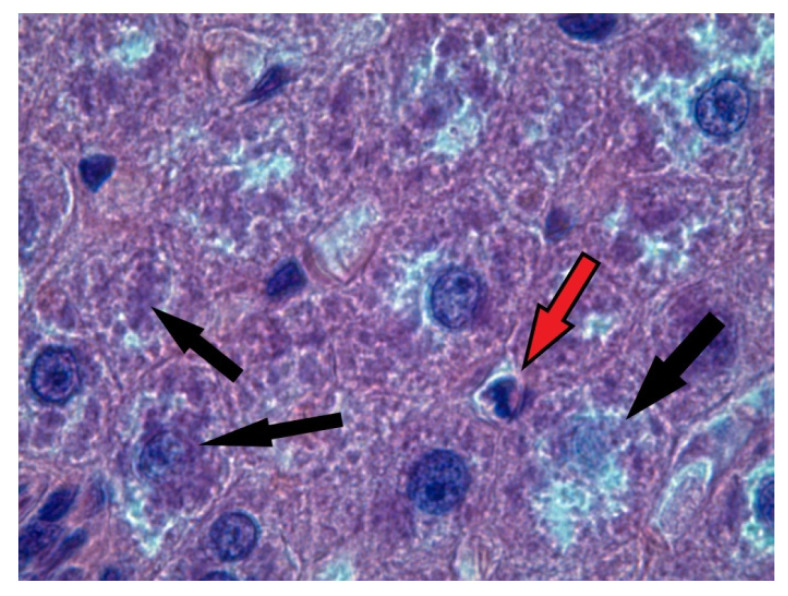
Liver of rat of EtOH + CL group. Hematoxylin and eosin staining, ×1000. Red arrow indicates hepatocyte in a state of apoptosis; black arrows—hepatocytes in a state of necrosis.

The morphological pattern of the liver in the rats of the control group corresponded to the age norm, i.e., the liver had a preserved structure of hepatic cords, composed of polygonal hepatocytes with a rounded, centrally located nuclei, without signs of dystrophic changes and necrosis. In the liver of rats of all of the experimental groups, a significant number of vacuole-containing hepatocytes was found (Figure 1). The control staining with Sudan-III verified the presence of lipid drops in the cytoplasm of the hepatocytes in this group, which indicates the beginning of the development of fatty degeneration of the liver. At the same time, we observed both single cells in a state of apoptosis and groups of hepatocytes in a state of necrosis (Figure 2).

In the liver of rats of the control group, a number of hepatocytes containing lipid drops were 2.40 ± 0.22%, thus, the steatosis grade was zero. In the liver of the EtOH and CL groups, a number of cells containing lipids were 16.30 and 10.35 times higher (39.20 ± 7.88% and 24.85 ± 5.96%); the steatosis grades were two and one, respectively. In the group EtOH + CL, steatosis grade was three; the proportion of cells in the state of fatty degeneration was 29.95 times higher than in control group (71.88 ± 8.84%).

### 2.2. Influence of Constant Lighting and CAI on the Micromorphometric Parameters of Hepatocytes of Rats

Three-week alcohol intoxication caused an elongation of the small and mean diameters of hepatocyte nuclei, a decrease in the nuclear contour index, and a significant increase in cell size, which led to a decrease in the NCR value.

Exposure to constant lighting during the same period of time led to significant differences from the norm in morphometric parameters. We found an increase in the area and volume of hepatocytes relative to the control, which caused a decrease in NCR. Moreover, with an increased area of nuclei, their long diameter also increased, and this change is reflected in a decrease in the elongation index.

The joint action of ethanol and constant illumination for three weeks also caused changes in the micromorphometric parameters of hepatocytes. The area, volume, and perimeter of the nucleus and NCR of hepatocytes decreased, as well as the length of the small diameter of the nucleus (Table 1).

### 2.3. Influence of Constant Lighting and CAI on Gene Expression

**Figure 3:**
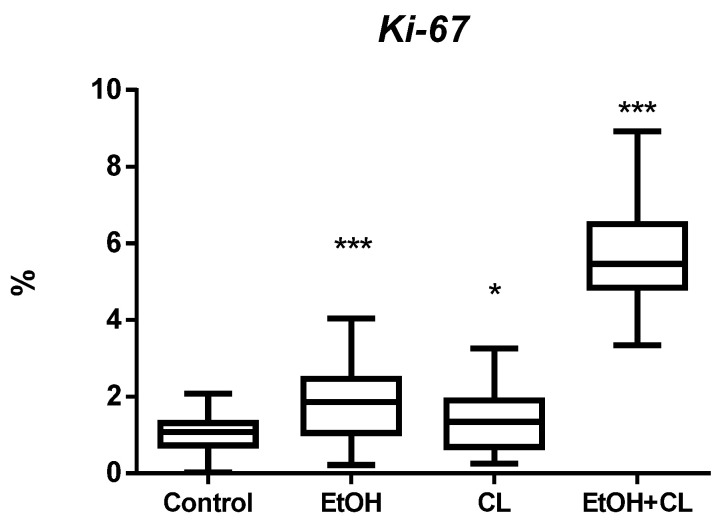
Levels of *Ki-67* expression in hepatocytes of rats. * (*p* ≤ 0.05); *** (*p* ≤ 0.0005)—in comparison with the parameters of animals of the control group.

**Figure 4:**
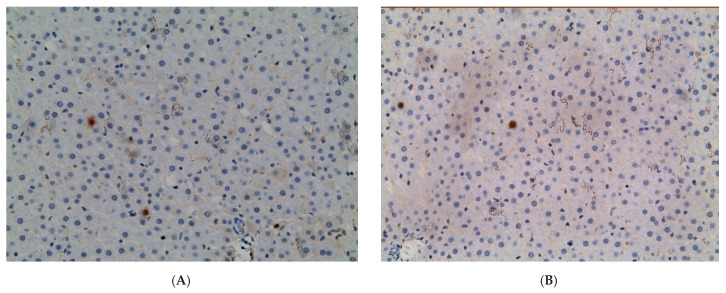

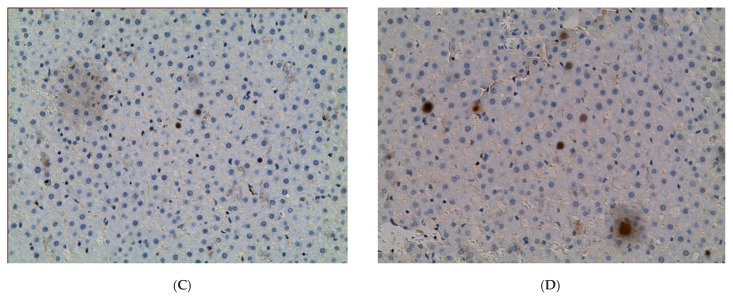
Expression of *Ki-67* in liver of rats. (**A**)—Control; (**B**)—EtOH; (**C**)—CL; (**D**)—EtOH + CL. ×200.

In the liver of control group, the proportion of *Ki-67+* hepatocytes was small—1.02 ± 0.40%. However, the value of this parameter was significantly higher in all experimental groups: 1.79 times (1.83 ± 0.87%) in EtOH group, 1.36 times (1.39 ± 0.78%) in CL, and 5.59 times (5.70 ± 1.30%) in EtOH +CL (Figure 3 and Figure 4).

**Figure 5:**
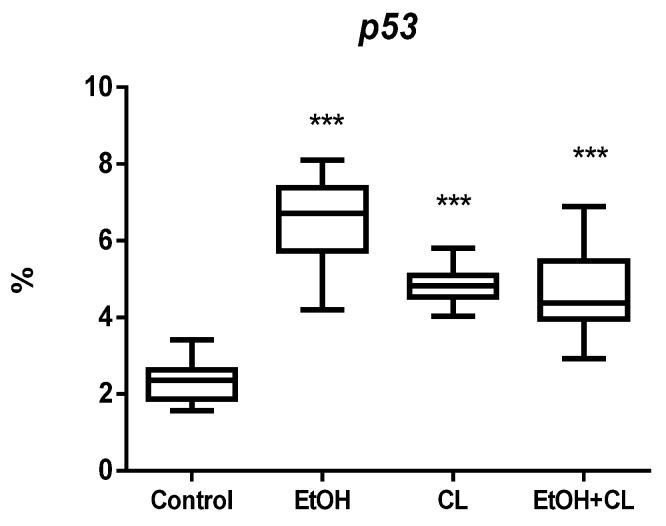
Levels of *p53* expression in hepatocytes of rats. *** (*p* ≤ 0.0005)—in comparison with the parameters of animals of the control group.

**Figure 6:**
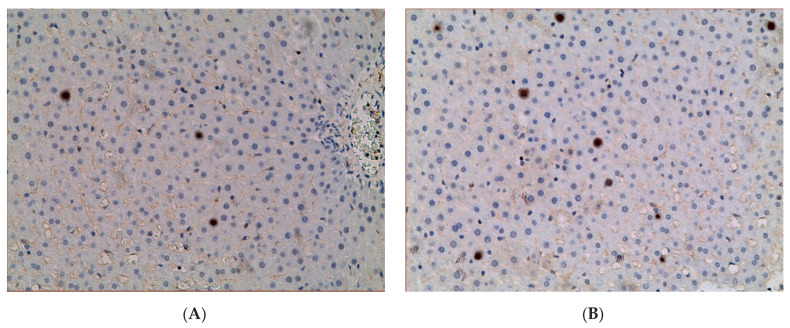

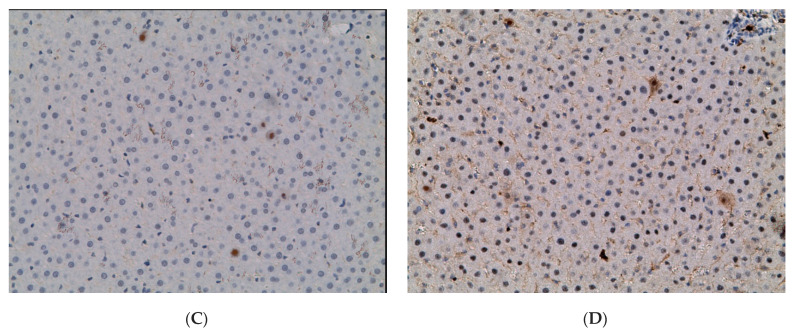
Expression of *p53* in liver of rats. (**A**)—Control; (**B**)—EtOH; (**C**)—CL; (**D**)—EtOH + CL. ×200.

In hepatocytes of animals of control group, *p53* expression was observed in 2.32 ± 0.50% of hepatocytes. It increased in animals of all experimental groups: 2.81 times higher in EtOH group (6.51 ± 0.94%), 2.06 times higher in CL (4.78 ± 0.63%), and 2.02 times higher (4.68 ± 0.95%) in EtOH + CL group (Figure 5 and Figure 6).

**Figure 7:**
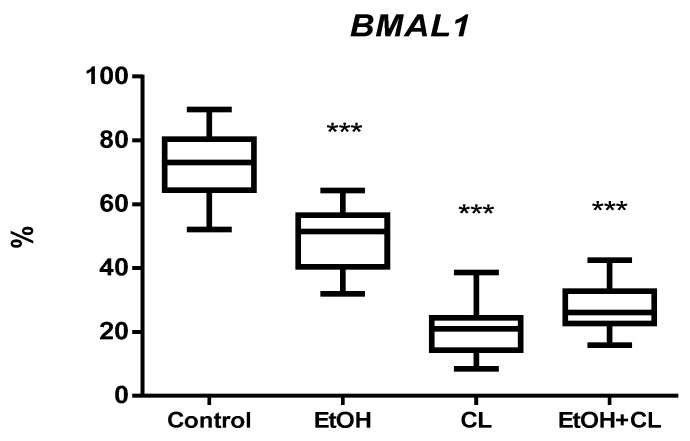
Levels of *BMAL1* expression in hepatocytes of rats. *** (*p* ≤ 0.0005)—in comparison with the parameters of animals of the control group.

**Figure 8:**
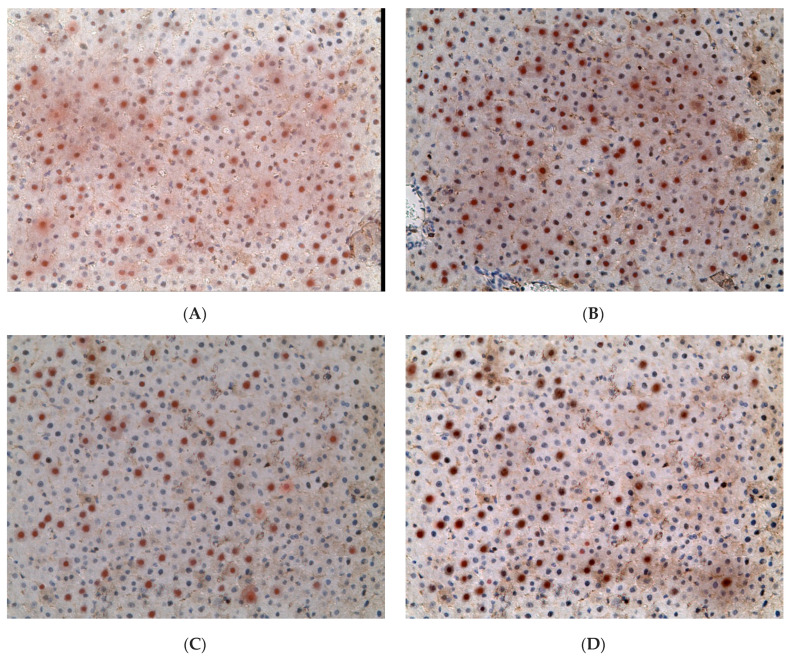
Expression of *BMAL1* in liver of rats. (**A**)—Control; (**B**)—EtOH; (**C**)—CL; (**D**)—EtOH + CL. ×200.

The proportion of *BMAL1*-positive hepatocytes in control group was 72.09 ± 9.63%. However, in females of the experimental groups there was a decrease in the proportion of *BMAL1+* cells. In animals of the EtOH group, their proportion was 1.47 times lower (48.91 ± 8.88%), in CL group—4.32 times lower (16.67 ± 6.84%), and 2.58 times lower (27.90 ± 6.91%) in rats of the EtOH + CL group (Figure 7 and Figure 8).

**Figure 9:**
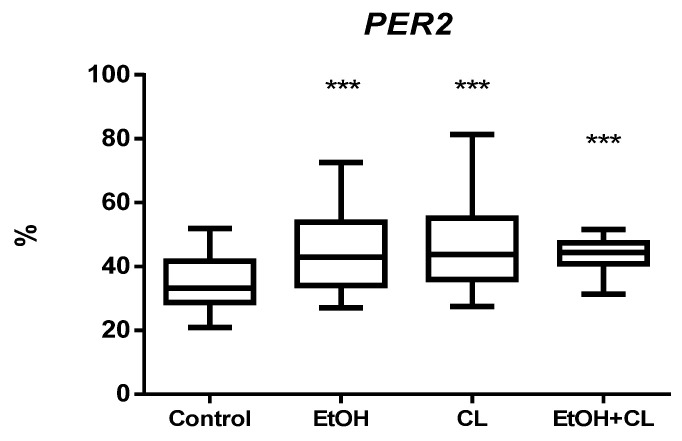
Levels of *PER2* expression in hepatocytes of rats. *** (*p* ≤ 0.0005)—in comparison with the parameters of animals of the control group.

**Figure 10:**
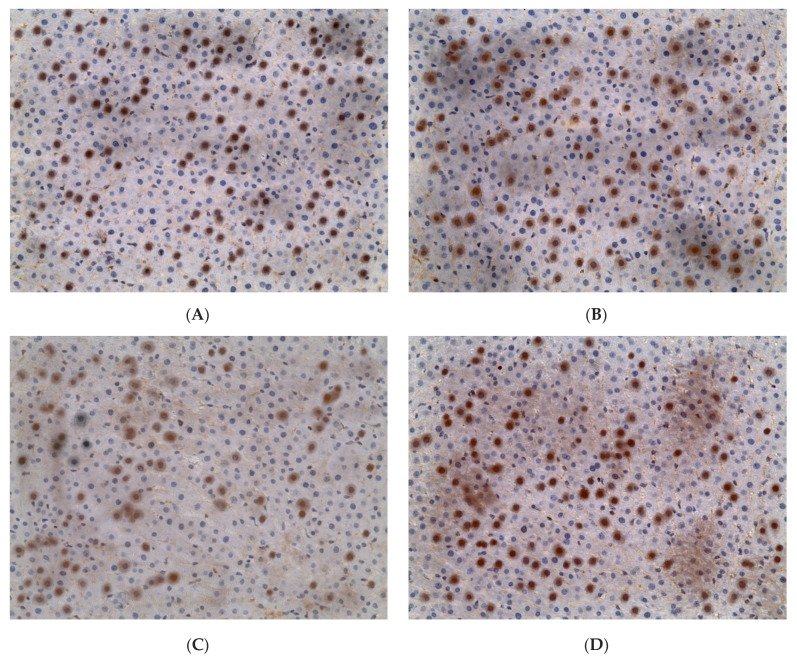
Expression of *PER2* in liver of rats. (**A**)—Control; (**B**)—EtOH; (**C**)—CL; (**D**)—EtOH + CL. ×200.

Expression of *PER2* in hepatocytes of rats in the control group was 34.99 ± 7.65%. In the liver of EtOH, CL, and EtOH + CL rats, the proportion of *PER2*-positive hepatocytes increased 1.27 times (44.53 ± 11.54%), 1.32 times (46.18 ± 13.05%), and 1.23 times (42.94 ± 6.33%), respectively (Figure 9 and Figure 10).

**Figure 11:**
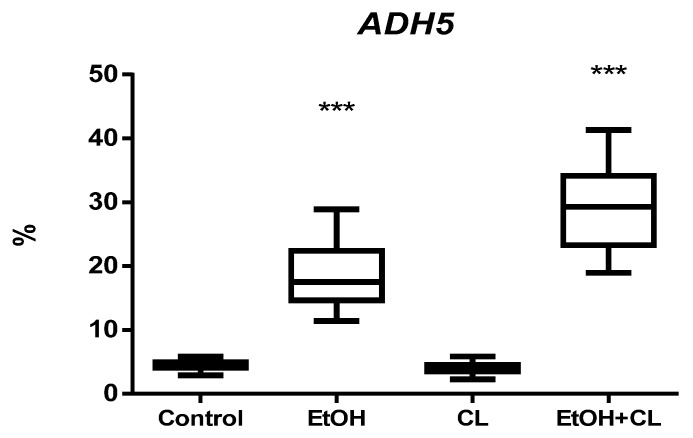
Levels of *ADH5* expression in hepatocytes of rats. *** (*p* ≤ 0.0005)—in comparison with the parameters of animals of the control group.

**Figure 12:**
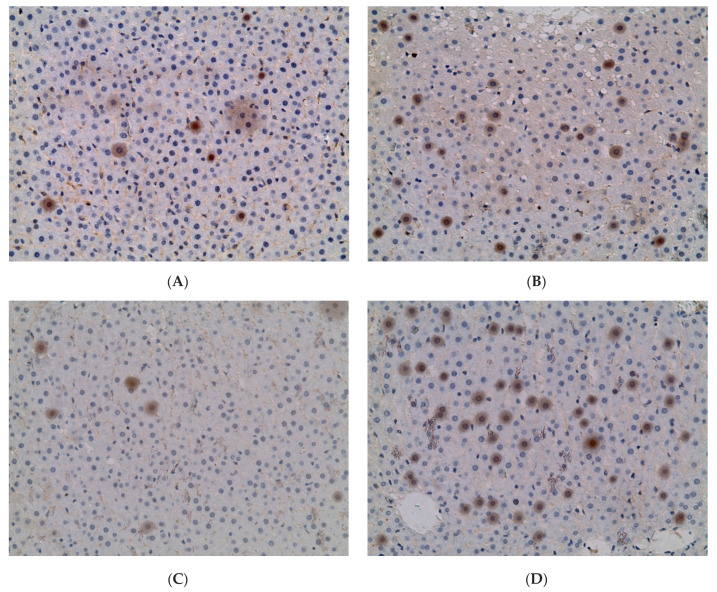
Expression of *ADH5* in liver of rats. (**A**)—Control; (**B**)—EtOH; (**C**)—CL; (**D**)—EtOH + CL. ×200.

*ADH5* expression was found in 4.33 ± 0.67% of hepatocytes of rats of the control group; it remained practically unchanged in the CL group, but increased 4.28 times (18.55 ± 4.72%) and 6.75 times (29.22 ± 5.94%) in the liver of females of groups EtOH and EtOH + CL (Figure 11 and Figure 12).

### 2.4. Influence of CAI and Constant Lighting on Circadian Dynamics of Morphometric Parameters of Hepatocytes

**Figure 13:**
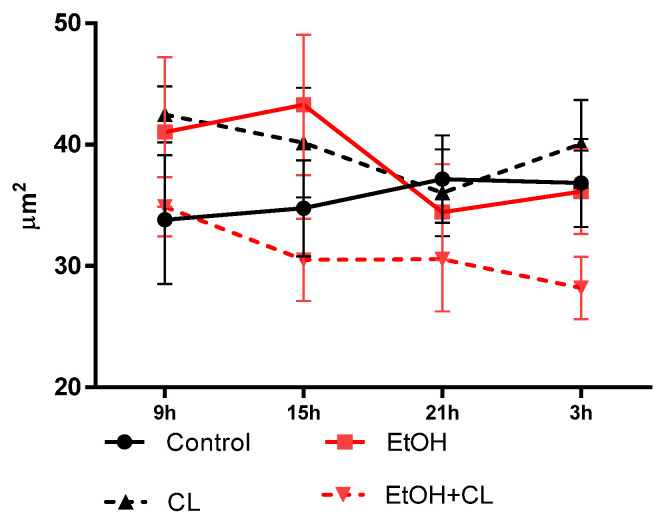
Daily dynamics of hepatocyte nuclei area of rats.

The minimum size of the nucleus in female rats under a fixed light regime (control) was observed at 09^00^, the maximum at 21^00^. Under CAI conditions, the minimum values of this parameter were noted at 21^00^ and the maximum at 15^00^. Under constant lighting, the extrema of the chronogram were opposite to those of the control, with a maximum at 9^00^ and a minimum at 21^00^. Under the combined action of ethanol and constant lighting with the same maximum, the minimum nuclei sizes were noted at 3^00^ (Figure 13).

In control group, the acrophase of rhythm was revealed at 23^39^ and amplitude of the rhythm made up 1.96 µm^2^. In CAI, the acrophase of the rhythm was observed at 12^08^ with 2.46 times higher amplitude than in control group (4.84 μm^2^). Under constant lighting and its combined action with CAI, acrophases of rhythms were detected in the morning hours, 9^04^ и and 10^51^ with amplitudes of 4.63 μm^2^ and 2.31 μm^2^–2.36 and 1.18 times higher than control, respectively (Table 2).

**Figure 14:**
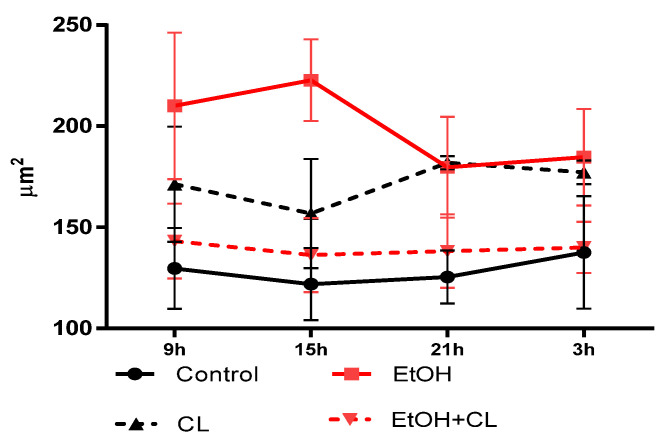
Daily dynamics of hepatocyte area of rats.

In females of the control group, hepatocytes reached their largest size by 3^00^, decreasing to a minimum at 15^00^. CAI at fixed light led to a shift of the maximum by 15^00^ and the minimum by 21^00^ h. Constant lighting caused opposite changes—the maximum was noted at 21^00^; the minimum at 15^00^. In animals of the EtOH + CL group, the largest sizes of hepatocytes were noted at 9^00^ with a minimum at 15^00^ (Figure 14).

In the control group, CR was characterized by an acrophase at 4^54^ and amplitude of 8.99 µm^2^, CAI caused a shift of the acrophase by 12^26^ and a 2.71 times increase in amplitude up to 24.34 µm^2^. CRs of hepatocyte area in groups CL and EtOH + CL were not significant (Table 2).

**Figure 15:**
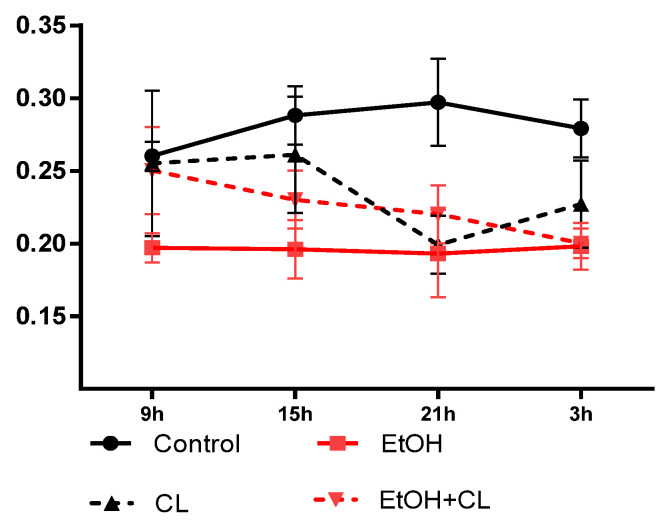
Daily dynamics of NCR of hepatocytes of rats.

In the liver of intact females, the maximum NCR was observed at 21^00^, and the minimum at 9^00^. In animals of the EtOH group, the maximum was shifted by 3^00^ h and the minimum was shifted by 21^00^ h. Under constant illumination with the same minimum, the maximum NCR was observed at 15^00^, and in rats of the EtOH + CL group, the maximum was observed at 9^00^ with a minimum at 3^00^ (Figure 15).

For the NCR rhythm in females of the control group, an acrophase was noted at 20^52^ and amplitude made up 0.020. The impact of CAI led to the destruction of the CR of NCR, constant illumination led to a displacement of the acrophase by 11^03^ and a 1.65 times increase in amplitude up to 0.033. In rats of the EtOH + CL group, acrophase was observed at 14^47^ with 1.17 times decreased amplitude (0.017) (Table 2).

### 2.5. Influence of Constant Illumination and CAI on the Organization of Circadian Rhythms of the Expression of the Studied Genes

**Figure 16:**
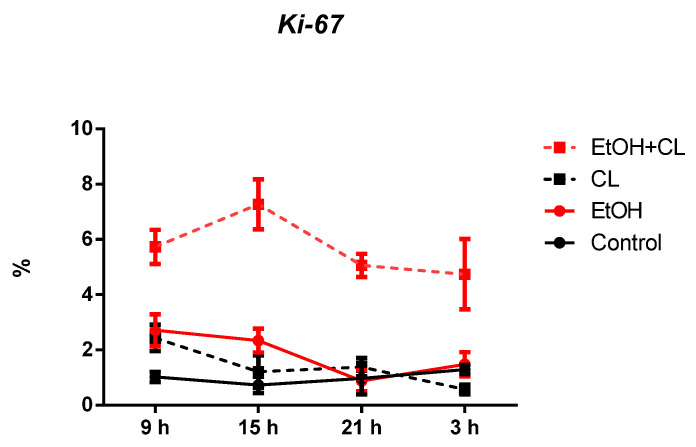
Daily dynamics of *Ki-67* expression in hepatocytes of rats.

In females of the control group, the maximum expression of *Ki-67* was noted at 3^00^ with a minimum at 15^00^. In the liver of animals of the EtOH group, the maximum occurred at 9^00^, the minimum–at 21^00^; in rats of the group CL, with the same maximum, the minimum expression was noted at 3^00^; in animals of the group EtOH + CL, the maximum was noted at 15^00^, the minimum–at 3^00^ (Figure 16).

Significant CRs were noted in the control—acrophase at 3^47^ with an amplitude of 0.29%—and in the liver of rats of groups EtOH and CL with acrophases of 10^40^ and 10^06^ and 3,6 times and 2.13 times increased amplitudes (1.04% and 0.62%), respectively (Table 3).

**Figure 17:**
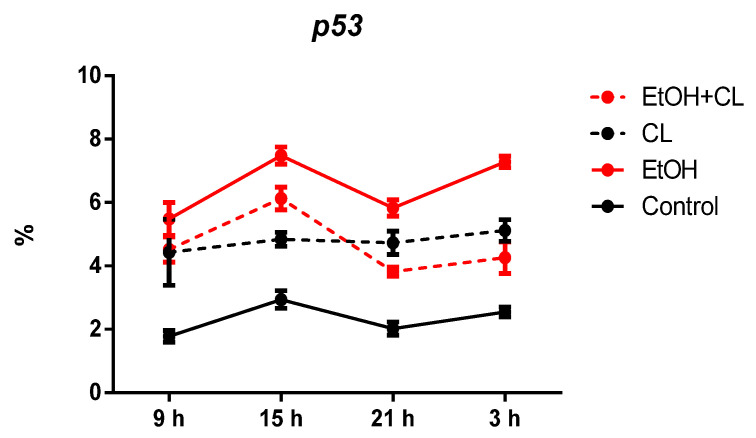
Daily dynamics of *p53* expression in hepatocytes of rats.

In hepatocytes of animals of control and EtOH groups, a maximum of *p53* expression was found at 15^00^ with a minimum at 9^00^. In group CL, the maximum expression of *p53* was noted at 3^00^ with a minimum at 9^00^, and in group EtOH + CL, a maximum at 15^00^ with a minimum at 21^00^ (Figure 17).

Significant CRs of this parameter were noted in the control group and in the EtOH and CL groups. In the control, the acrophase of the rhythm was noted at 17^06^ with an amplitude of 0.23%. In the group EtOH, the acrophase shifted by 18^54^ with a practically unchanged amplitude, and in the hepatocytes of rats of the group CL, an acrophase was noted at 11^19^ and the amplitude of the rhythm increased 7.17 times up to 1.65% (Table 3).

**Figure 18:**
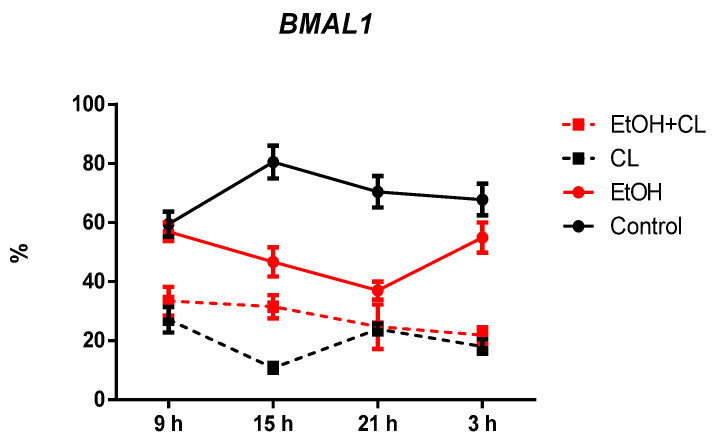
Daily dynamics of *BMAL1* expression in hepatocytes of rats.

Considering the daily dynamics of *BMAL1* expression in hepatocytes of rats of the control group, we found a maximum expression at 15^00^ and minimum–at 9^00^, and in groups EtOH, CL, and EtOH + CL, with the maximum at 9^00^, the minimum expression of *BMAL1* was found at 21^00^, 15^00^, and 3^00^, respectively (Figure 18).

CR of *BMAL1* expression in hepatocytes of rats of control group was characterized by an acrophase of 4^19^ and an amplitude of 11.14%, in cells of group EtOH, 7^30^ and 10.78% (1.03 times lower), and in cells of group CL, by acrophase of 4^56^ and an amplitude of 3.95% (2.82 times lower) (Table 3).

**Figure 19:**
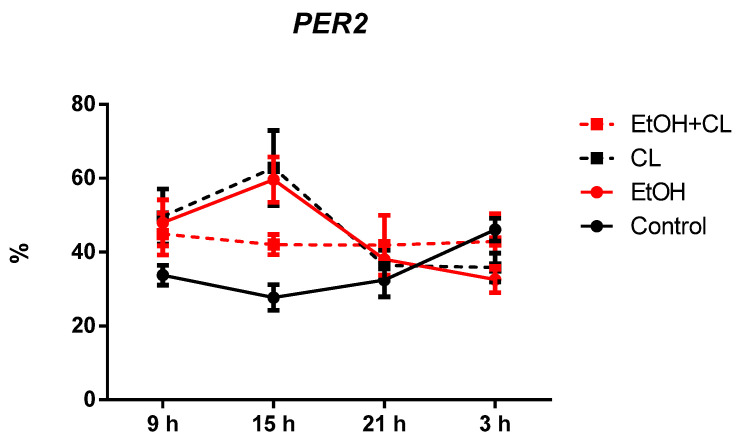
Daily dynamics of *PER2* expression in hepatocytes of rats.

In intact rats, the extremum points of the *PER2* expression rhythm were characterized by a maximum at 3^00^ with a minimum at 15^00^. In animals of EtOH and CL groups, the maximum was noted at 15^00^, the minimum at 3^00^, and in the rats of the group EtOH + CL, both extrema were shifted to the previous time points (Figure 19).

Significant CRs were noted in the control group—acrophase at 3^16^ and amplitude of 9.22%—and in EtOH and CL groups, with similar parameters—acrophases at 13^39^ and 13^16^ and an amplitude of 14.41% and 15.03% (1.56 and 1.63 times higher than in control group), respectively (Table 3).

**Figure 20:**
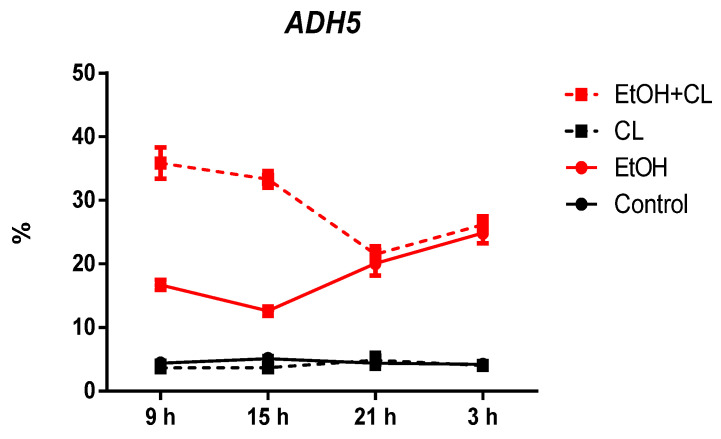
Diurnal dynamics of *ADH5* expression in hepatocytes of rats.

In rats of the control group, the maximum of *ADH5* expression was noted at 15^00^, the minimum at 3^00^; in animals of the EtOH group the picture was opposite to the control. In the CL group, the maximum was noted at 21^00^ with a minimum at 9^00^; the parameters of the EtOH + CL group were opposite to those of CL (Figure 20).

Cosinor analysis revealed that CRs of this parameter were present in all groups of female rats. In the control group, it was characterized by an acrophase at 15^01^ and an amplitude of 0.46%; in group EtOH, acrophase occurred at 1^56^ with 13.78 times higher amplitude (6.34%); in group CL, acrophase is noted at 22^05^ with 1.34 times increased amplitude (0.62%); in group EtOH + CL, acrophase was noted at 12^19^ and the amplitude of the rhythm increased 16.54 times (7.61%) (Table 3).

## 3. Discussion

The study of pathomorphological changes in the liver of female rats showed that the influence of two factors—constant lighting and chronic alcohol intoxication—led to significant disturbances in the morphofunctional condition of this organ within three weeks. These changes were manifested in the development of the fatty degeneration of the liver, which is most pronounced at combined action of CAI and constant lighting.

Our previous studies [55,56,57,58] showed that both constant illumination and CAI have a significant effect on the morphological and functional integrity of the liver of male rats, which manifests itself primarily in the development of fatty degeneration and steatohepatitis in them. However, our study revealed a number of significant gender differences in the response of hepatocytes to the combined and separate effects of these factors. So, in males, the influence of alcohol under a normal (fixed) light regime provoked the occurrence of fatty degeneration of the liver, and the same factor in combination with constant illumination led to the development of hepatitis; in some animals, there were already signs of liver cirrhosis. In females of all experimental groups, under the same conditions, we noted only fatty degeneration.

There are a number of common features in pathogenesis of alcoholic liver disease (ALD) and NAFLD [68]. Both ALD and NAFLD are based on steatosis, which is considered a benign and reversible condition. It is shown that one of the contributing factors in the development of NAFLD is the disruption of the day/night cycle [69].

It was also found that CAI and dark deprivation cause a number of changes in the micromorphometric parameters of hepatocytes. In particular, in animals of the EtOH and CL groups, hypertrophy of hepatocytes was noted. The NCR decreased in rats of all groups. The greatest changes, manifested in a decrease in the size of the nucleus, were noted in the liver of rats of the EtOH + CL group. At the same time, in males under the same conditions, a three-week CAI did not cause changes in the studied micromorphometric parameters.

According to some reports, hepatocyte hypertrophy can be observed during polyploidization of nuclei and during the formation of binuclear cells. In turn, an increase in the linear dimensions of hepatocytes after three weeks under constant illumination is the result of an intensification of their functional activity. Increase in the areas of hepatocytes is also associated with an increase in levels of stress hormones. Such changes are described for the liver under conditions of chronic stress, such as the 21-day darkness deprivation [70,71].

The development of small-drop fatty degeneration in hepatocytes under stress is a described phenomenon; it correlates with the duration of stress exposure [72], being associated with an increase in the level of adrenal cortex hormones, primarily glucocorticoids. This, in turn, causes an increase in the expression of serotonin 5-HT2A and 5-HT2B receptors and tryptophan hydroxylase 1, as well as in the synthesis of serotonin [73]. The accumulation of lipid droplets by hepatocytes under stress is accompanied by an increase in lipolysis gene expression and β-oxidation of fatty acids [74].

Increased expression of *Ki-67* and *p53* indicates intensification of proliferation and apoptosis processes in the liver of rats of all experimental groups [75]. The increase in the expression of these genes in our study is least pronounced in animals of the CL group. At the same time, the expression of *p53* increased to a greater extent in rats of the EtOH group, and the expression *Ki-67* in animals of the EtOH + CL group. Decrease in the expression of *BMAL1* and increase in the expression of its antagonist *PER2* served as a confirmation of the chronodestructive effects of both constant lighting and ethanol. The expression of *ADH5,* as expected, was higher in the hepatocytes of rats that consumed ethanol.

Numerous studies indicate that lipid metabolism disorders can enhance the expression of the proapoptotic *p53* protein [76]. Apoptosis plays a significant role in the pathogenesis of non-alcoholic hepatosteatosis, but the mechanisms of its intensification have not been fully elucidated. There is an opinion that the increased expression of the *p53* protein, indicating the intensification of apoptosis in hepatocytes, can be induced by the activation of inflammatory stimuli and oxidative stress [77]. It can be assumed that increased expression of this protein is only one link among numerous factors involved in the mechanism of hepatosteatosis development.

There is an opinion that in disorders of lipid metabolism of the liver, an increase in *p53* expression is accompanied by a decrease in the intensity of proliferation [78]. However, in males exposed to the combined influence of CAI and constant illumination, an increase in *Ki-67* expression occurred, and in females this was observed in all experimental groups. Studies by S. Stöppeler [79] showed increased expression of *Ki-67* in experimental steatosis in rats, with females having a higher expression level than males. It was assumed that this is due to the work of some genes of receptors of *VGF* (*VGF nerve growth factor inducible) 1* and *2* (*FLT1, FLK1*), which demonstrate a higher expression profile in females. These changes in gene expression were in agreement with higher proliferation rates of females. *VGF*-mediated signaling can protect hepatocytes and improve the impaired regeneration of steatotic liver [80].

Thus, with the same proapoptotic activity in hepatocytes, females showed regenerative activity already under the separate influence of the studied chronodestructors.

The decrease in the expression level of *BMAL1* as a result of CAI, which we found, is confirmed by studies [81]. It is noteworthy that the *BMAL1* protein plays an important role in the protection of hepatocytes in alcoholic liver disease [82]. At the same time, an increase in the expression of the *PER* family genes in the liver was described in response to alcohol consumption [83]. Changes in the expression of *BMAL1* and *PER2* during long-term constant illumination were probably the result of long-term melatonin deficiency [84,85].

In our studies, there was an increase in both *p53* and *PER2* in the liver of animals of all experimental groups. There are some data that *PER2* may directly regulate *p53* activity: inactivation of *PER2* by mutation delayed *p53* accumulation after ionizing radiation, sensitizing mice to both cancer development and death [86]. Supporting these data, it was showed that high levels of *PER2* in cancer cell lines and glioma xenografts correlated with increased p53 induction and apoptosis [87].

The mechanisms of interaction between *PER2*, *p53*, and *MDM2* (*Mouse double minute 2 homolog*) are not fully understood to date. It is proved that interrelation between *p53* and *PER2* is bidirectional, as *p53* can influence *PER2* both at the gene expression and protein level. Under unstressed conditions, *PER2* and *p53* form a stable complex in the cytosol and, along with *MDM2*, a trimeric complex in the nucleus. Association of *PER2* to the C-terminus end of *p53* prevents *MDM2* (*Mouse double minute 2 homolog)* mediated ubiquitylation and degradation of *p53* as well as *p53*’s transcriptional activation. *p53* can antagonize *PER2* expression by directly binding to the *PER2* promoter and blocking CLOCK-BMAL1 transactivation of the gene [88].

Gallego and Virshup (2007) [89] proposed a hypothesis that *PER2* may exist in two pools: one bound to *p53* and one bound to *CRY* and *CK1ε* for control of circadian rhythm and subsequent degradation. It was also assumed that in stress conditions, *p53* induction will radically alter the circadian clock through its modulation of *PER2*, which may perhaps be an adaptive pro-survival process [90].

CRs of morphometric parameters undergo significant changes under the influence of CAI and constant lighting. The most stable is the CR of the area of nuclei, which was preserved in a rearranged form in the hepatocytes of rats of all experimental groups. The CR of the hepatocyte area was destroyed under constant illumination, and the NCR rhythm was destroyed only at CAI.

We did not find an explanation for this fact in the available literature. It can be assumed that both in the case of the influence of ethanol on the SCN and the pineal gland, and in the case of the deficiency of pineal melatonin due to dark deprivation, the role of pacemaker was played by some other structure or process. Intracellular processes associated with the regulation of the rhythms of the molecules that form the cytoskeleton of the cell can be proposed as candidates for this role [91].

In females, the destruction of the CRs of expression of *p53* and *Ki-67* was observed only in rats that were under the combined influence of CAI and constant illumination, while in males, CRs of expression of these genes was noted only in the control and in animals under CAI conditions with a changeable light regime, albeit in a rearranged form.

Similarly, we found that in females, the CRs of expression of *BMAL1* and *PER2* were present in hepatocytes of all groups, except rats which were under the combined influence of CAI and constant lighting, while in males it was destroyed both under the separate action of CAI and under the action of CAI combined with constant illumination.

The rhythm of *ADH5* expression in hepatocytes of females was preserved in all groups; in males it is destroyed only under constant illumination.

Thus, it can be assumed that the CRs of expression of *Ki-67* and *p53* are more affected by the light regime, while the violation of the clock gene expression CR is largely caused by the influence of ethanol.

At the same time, it is necessary to note the high stability of the studied CRs in females. In males, the CRs of *Ki-67* and *p53* expression were destroyed under constant illumination and the combined action of CAI and dark deprivation, and the rhythms of the clock genes were destroyed under both separate action of CAI and its complex with light. In females, only the combined action of chronodestructors caused the destruction of these CRs, and the rhythm of *ADH5* expression was preserved in animals of all groups.

Sex differences in the circadian system in mammals arise already at the level of SCN, whose neurons have estrogen and androgen receptors. Even afferent inputs to the SCN, including the retinohypothalamic tract, are characterized by sexual characteristics [92].

One of the probable explanations for intersexual differences in the circadian organization is that the formation of the structure of CRs of an organism can occur during the prenatal period or during the perinatal period of sexual differentiation of the brain [93].

For example, from the moment of birth to the end of the third week of postnatal ontogenesis, male mice showed a significantly higher expression of androgen receptors in the SCN than female mice [94]. The mechanism of this gender difference remains unclear, but it does not depend on the regulation of sex hormones.

At the same time, it is shown that SCN neurons in adult mammals have a small amount of estrogen receptors, but are rich in androgen receptors. They are localized mainly on neurons of the ventrolateral part of the SCN that secrete gastrin-releasing peptide (*GRP*); in addition, terminals of retinal axons are projected onto the same neurons, which can cause depolarization of these cells and cause expression of the *PER* gene [95].

Androgen receptor-positive cells carry out light-induced *FOS (FOS Proto-Oncogene, AP-1 Transcription Factor Subunit)* expression; this process can be attenuated by gonadectomy and restored by the non-aromatizing androgen dihydrotestosterone. Thus, both light impulses from the external environment and male sex hormones affect the same population of SCN neurons, and, in turn, the rhythm of testosterone secretion is regulated by this brain structure, forming a neuroendocrine feedback loop in the circadian system [96].

In females, neurons containing estrogen receptors are located predominantly outside the SCN, but in structures with a large number of projections into it [97]. For example, estradiol modulates light-induced *FOS* expression of serotonergic neurons in the dorsal raphe nucleus, which has afferent connections with SCN [98].

Morphological sex features of the SCN structure are described for both humans and animals. For example, electron microscopic analysis shows that in the SCN of male rats, the number of synapses in neurons is higher than in females, and neuron nuclei contain more nucleoli [99]. In gerbils, the volume of the SCN is sexually dimorphic, as is the organization of the astroglia [100]. In humans, the volume and length of the rostrocaudal axis of the SCN are greater in women [101]. Sex differences in electrical activity coincide with the above anatomical features; the transmission and processing of sensory information through sex-dimorphic neural networks also differ [102]. Sexual differences in circadian rhythm may also occur in structures that receive information from the SCN. For example, in the hypothalamic–pituitary–adrenal system (HPAS), the circadian rhythm of glucocorticoids is well-studied, which, in addition to classical functions, are involved in the regulation of the expression of some clock genes (in cells of the myocardium, liver, and kidneys) [103,104]. Sexual dimorphism in the CRs of synthesis and secretion of hormones of adrenal cortex, which determine the rhythm of a number of physiological processes, is described [105].

In addition, gender differences in CRs of melatonin are described; this hormone has a wide range of effects on functions of organisms and is involved in the regulation of a number of circadian rhythms. Thus, it was found that in women, the CR’s amplitude of melatonin is significantly higher, the amplitude of the CR of temperature is lower, and the synchronization of daily rhythms of body temperature and melatonin secretion in conditions of isolation from external factors proceeds faster than in men [106].

Sexual characteristics were noted in the functioning of the hypothalamic–pituitary–adrenal system in general and the CRs controlled by it. Corticosteroids, which normally have strict CRs, in addition to their traditional functions (participation in stress reactions, energy metabolism, etc.), perform the function of secondary oscillators, having the ability to shift the expression of some clock genes in peripheral organs, including the liver [107].

## 4. Materials and Methods

### 4.1. Object of Study

This study was conducted on 160 female rats of Wistar outbred stock at an age of 6 months, with a body weight of 350 g. Animals were taken from the “Stolbovaya” affiliate of the FSBIS Scientific Center for Biomedical Technologies of the Federal Medical and Biological Agency. Initially, the animals were kept in natural lighting, at a temperature of 20–22 °C and a relative humidity of 60–70%. The rats had free access to drinking water and briquetted food. Keeping of animals and experiments were performed in accordance with the European Convention for the Protection of Vertebrate Animals used for Experimental and other Scientific Purposes (Strasbourg, 18 March 1986). This research was approved by the Bioethical Committee of the Federal State Budgetary Scientific Institution “Research Institute of Human Morphology”, protocols No. 27/3 (11.10.2021), No. 34 (10) (14.03. 2022).

### 4.2. Design of Study

Rats were divided into 4 equal groups. To model the CAI, we used a 15% aqueous solution of ethanol [108].

Control group (n = 40) (control) was kept under fixed light regime (light:dark/10:14 h with lights on at 8:00 and off at 18:00).

First group (n = 40) (EtOH) was kept at the same conditions as control, but a 15% aqueous ethanol solution was offered daily as a drink ad libitum instead of water.

Second group (n = 40) (CL) was kept under the regime of constant lighting.

Third group (n = 40) (EtOH + CL) was kept under the regime of constant light and received as a drink a 15% aqueous solution of ethanol ad libitum.

The criterion for selecting rats for the study, along with the absence of visible abnormalities in condition and behavior, was the initial preference for a 15% solution of ethyl alcohol in comparison with tap water. For this, a preliminary experiment was carried out for 3 days in individual cages with free access to both liquids.

During the experiment, the volume of the drunken ethanol solution was determined daily, and then, the mass of alcohol per 1 kg of body weight was calculated. On average, the animals drank 15.48 ± 1.28 mL/day, which in terms of absolute ethanol is 7 g/kg of body weight.

Euthanasia was carried out three weeks after the start of the experiment in a carbon dioxide chamber equipped with a device for the upper gas supply (100% CO_2_) at 9:00, 15:00, 21:00, and 3:00. The chamber volume was filled with gas at a rate of 20% per minute to avoid dyspnea and pain in animals. After sacrifice, the evisceration was performed.

### 4.3. Morphological, Morphometric, and Histochemical Methods

The liver was fixed in 10% neutral buffered formalin with further passage through alcohols of increasing concentration (50%, 60%, 70%, 80%, and 96%) and xylol, followed by pouring into Histomix histological medium (BioVitrum, Russia). When conducting studies of organs embedded in paraffin, serial sections with a thickness of 5–6 μm were prepared. Histological sections were made on the sliding microtome Leica SM2010 R (Germany). Hematoxylin and eosin staining was carried out according to the standard technique [109]. Stained sections were put in a BioMount mounting medium (BioVitrum, Russia).

Fragments of the liver were frozen for subsequent histological examination, using a freezing table for the MFT-01 “Unicon” microtome; serial frozen sections with a thickness of 6–8 μm were prepared. To confirm the presence of fatty degeneration, standard staining of frozen sections with a solution of Sudan-III in 70% ethyl alcohol was performed [110]. Steatosis (percentage of hepatocytes containing lipid droplets) was scored using the non-alcoholic fatty liver disease (NAFLD) activity scoring (NAS) protocol. While the NAS protocol is not intended for AD, we applied this system to assign a histopathology score to cases in this experimental animal study. Steatosis was scored as: 0, <5%; 1, 5–33%; 2, >34–65%; 3, >66% of hepatocytes containing lipid droplets [111]. Microscopy of histological preparations was performed using a Leica DM 2500 microscope with use of a Leica DFC 290 digital camera (Germany). From each studied preparation, 10 digital images of randomly selected visual fields were taken at a magnification of ×200, ×400, and ×1000, with the use of which karyo- and cytometry were subsequently carried out. In morphometric studies, the Fiji software package, a program built on the basis of ImageJ v2 with appropriate plugins, was used [112]. The measurements were carried out in micrometers after preliminary geometric calibration on an object-micrometer scale digitized with the same magnification. Micromorphometry was performed only for mononuclear interphase hepatocytes without signs of pathological changes.

With use of «ImageJ», the cross-sectional area of nuclei of hepatocytes (area of nuclei, S_n_), small (d) and long (D) diameters of nuclei, perimeter of nuclei (P_n_), cross-sectional area of hepatocytes (area of cell, S_cell_), and small (a) and long (b) diameters of hepatocytes, were determined.

The several parameters were calculated with use of appropriate formulas [113].

Nuclear-cytoplasmic ratio of hepatocytes was calculated by the formula: NCR = S_n_/S_c_, where: S_n_—area of nucleus of cell; S_c_—area of cytoplasm.

Mean diameter of nuclei was calculated by the formula: M = (D + d)/2, where D—long diameter; d—small diameter [113].

Volume of nuclei was calculated by the formula: V_n_ = 0.523 M^3^, where M—mean diameter of nuclei.

Volume of cells was calculated by the formula: V_c_ = 0.523 M^3^, where M—mean diameter of cells.

The nucleus volume to nucleus area ratio (V/A coefficient) was calculated by the formula: V_n_/A_n_, where V_n_ is the mean volume of nuclei; A_n_—mean area of nuclei.

Elongation index of nucleus was calculated by the formula: EI = D/d, where D—long diameter; d—small diameter.

For calculation of coefficient of form, the formula was used: CF = 4 × π × S_n_/P_n_^2^, where S_n_—area of nucleus; P_n_—perimeter of nucleus.

Contour index of nucleus, which reflects the topography of its surface, was determined by formula: CI= P_n_/√ S_n_, where S_n_—the area of nucleus; P_n_—perimeter of nucleus.

### 4.4. Immunohistochemical Methods

To carry out immunohistochemical reactions, liver sections were dewaxed, rehydrated, and treated with 3% hydrogen peroxide solution to block endogenous peroxidase. Then, the slices were put in the «Ultra V Block» (Thermo Fisher Scientific; Waltham, MA, USA) solution; the antigens were previously unmasked by boiling in citrate buffer (pH 6.0). Immunohistochemical reactions with primary antibodies were performed [114].

The following antibodies were used:

Ki-67–Rabbit polyclonal (Cloud-Clone Corp., Houston, TX, USA), 1:300;
*PER2*–Rabbit polyclonal (Cloud-Clone Corp., Houston, TX, USA), 1:200;
*BMAL1*–Rabbit polyclonal (Cloud-Clone Corp., Houston, TX, USA), 1:200;
*p53*–Rabbit polyclonal (Cloud-Clone Corp., Houston, TX, USA), 1:200;
*ADH5*–Rabbit polyclonal (Cloud-Clone Corp., Houston, TX, USA), 1:300.

Sections were incubated with antibodies for 60 min at room temperature. The UltraVision Quanto Detection System (Thermo Fisher Scientific; Waltham, MA, USA) set was used as a detection system.

Reactions with replacement of primary antibodies with phosphate buffer solution served as control.

After the sections acquired a blue hue, the slides were removed, dehydrated in alcohols of ascending concentration and xylene according to the standard scheme, and embedded in the BioMount mounting medium (BioVitrum, St Petersburg, Russia).

The results of immunohistochemical reaction were appreciated by the proportion of stained cells or cell nuclei (depending on the localization of the antigen) in relation to the total number of hepatocytes. Two investigators reviewed and scored slides independently by estimating the percentage of hepatocytes showing characteristic staining. Percentage of stained cells was estimated in 4 fields of view at ×400 magnification. The expression of studied genes was assessed by counting the percentage of positive cells over the total cells in each slide and expressed as percentages of positive cells (0–100%) [115].

### 4.5. Methods of Statistical Processing

The obtained data were analyzed using the “GraphPad Prism 6.0” software by calculating the mean values, standard deviation, and mean error of the arithmetic mean. Numerical rows characterizing the daily fluctuations of the studied physiological rhythms of animals were subjected to mathematical processing, on the basis of which the group chronograms were drawn. We studied the shape of the chronograms and calculated the average daily values. The statistical difference was determined using the Kruskal–Wallis test. Differences were considered statistically significant at *p* < 0.05.

Cosinor analysis with the application of CosinorEllipse2006-1.1 program (certificate of state registration No. 2006611345, Siberian State University of Physical Culture and Sports, Omsk, Russia) was used to calculate the amplitude and acrophase of CRs.

Cosinor analysis was assigned to analyze wave processes and process chronobiological data. The presence of a reliable circadian rhythm was determined, as well as its acrophase and amplitude. The output information of the cosinor analysis was the main parameter of the rhythms: mesor, i.e., the value of the average level of the sinusoid (h), the amplitude of the sinusoid (A), and the acrophase (Phi), that is the time of the onset of the maximum of the function. Mesor coincides in magnitude with the average daily value of the investigated function. Acrophase is a measure of the peak time of total rhythmic variability over a 24-h period, i.e., the time of the onset of the maximum of the function. The amplitude corresponds to half of the total rhythmic variability in the cycle. Acrophase is expressed in hours; amplitude values are expressed in the same units as the studied variables [116].

## 5. Conclusions

The explanation of the effects of dark deprivation, CAI, and their combined action, as well as their intersexual differences, that we discovered, is rather complicated and requires further study. It remains unclear what is the leading factor in the effect of alcohol on the CRs of the expression of the studied genes: a direct effect on the hepatocyte, or mediated through the SCN, the pineal gland, and secondary oscillators, or whether there is an effect along all pathways. It is also necessary to continue research on the effect of constant illumination on these CRs, since dark deprivation, in addition to its effect on SCN, causes a decrease in the level of pineal melatonin, which is actively involved in the organization of the CRs and is an adaptogen with a wide action profile.

## Figures and Tables

**Table 1:** Results of micromorphometric studies of hepatocytes of rats.

	Control	EtOH	CL	EtOH + CL
**Cross-sectional area of nuclei, µm^2^**	36.54 ± 4.26	38.72 ± 6.0	39.69 ± 4.17 *	31.03 ± 3.98 **
**Volume of nuclei, µm^3^**	130.80 ± 28.73	149.40 ± 39.79	144.20 ± 50.23	108.30 ± 20.36
**Perimeter of nuclei, µm**	24.83 ± 2.46	23.59 ± 1.98	26.28 ± 2.05	21.38 ± 1.28 ***
**Nucleus volume to nucleus area ratio (V/A coefficient)**	3.70 ± 0.85	3.82 ± 0.48	3.61 ± 1.11	3.51 ± 0.56
**Long diameter of nuclei, µm**	6.78 ± 0.40	6.90 ± 0.54	7.23 ± 0.63 **	6.44 ± 0.39
**Small diameter of nuclei, µm**	5.76 ± 0.69	6.36 ± 0.90 **	5.81 ± 1.53	5.29 ± 0.44
**Mean diameter of nuclei, µm**	6.26 ± 0.45	6.63 ± 0.55 *	6.52 ± 0.71	5.85 ± 0.37 *
**Elongation index of nucleus**	1.19 ± 0.13	1.10 ± 0.15	1.39 ± 0.64 **	1.22 ± 0.09
**Contour index of nucleus**	4.19 ± 0.56	3.80 ± 0.13 *	4.19 ± 0.39	3.85 ± 0.06
**Coefficient of form of nucleus**	0.75 ± 0.16	0.87 ± 0.07	0.73 ± 0.12	0.85 ± 0.02
**Cell area, µm^2^**	128.60 ± 20.51	199.30 ± 31.57 ***	171.8 ± 21.36 ***	139.5 ± 16.62
**Cell volume, µm^3^**	1118.0 ± 268.80	2050.0 ± 494.1 ***	1571.4 ± 302.7 ***	1196.0 ± 214.1
**NCR**	0.29 ± 0.07	0.20 ± 0.02 ***	0.24 ± 0.04 ***	0.22 ± 0.026 ***

Note: hereinafter * (*p* ≤ 0.05); ** (*p* ≤ 0.005); *** (*p* ≤ 0.0005)—in comparison with the parameters of animals of the control group.

**Table 2:** Results of the cosinor analysis of the daily dynamics of the micromorphometric parameters of hepatocytes of rats.

Group	Acrophase	Amplitude
**Area of Nuclei of Hepatocytes, μm^2^**		
**Control**	23^39^	1.96
**EtOH**	12^08^	4.84
**CL**	9^04^	4.63
**EtOH + CL**	10^51^	2.31
**Area of Hepatocytes, μm^2^**		
**Control**	4^54^	8.99
**EtOH**	12^26^	22.34
**CL**	Not significant CR	
**EtOH + CL**	Not significant CR	
**NCR**		
**Control**	20^52^	0.02
**EtOH**	Not significant CR	
**CL**	11^03^	0.033
**EtOH + CL**	14^57^	0.017

**Table 3:** Results of the cosinor analysis of the daily dynamics of the expression of the studied genes.

Group	Acrophase	Amplitude
***Ki-67*, %**		
Control	3^47^	0.29
EtOH	10^40^	1.04
CL	10^06^	0.62
EtOH + CL	Not significant CR	
***p53*, %**		
Control	17^06^	0.23
EtOH	18^54^	0.24
CL	11^19^	1.65
EtOH + CL	Not significant CR	
***BMAL1*, %**		
Control	4^19^	11.14
EtOH	7^30^	10.78
CL	4^56^	3.95
EtOH + CL	Not significant CR	
***PER2*, %**		
Control	3^16^	9.22
EtOH	13^39^	14.41
CL	13^16^	15.03
EtOH + CL	Not significant CR	
***ADH5*, %**		
Control	15^01^	0.46
EtOH	1^56^	6.34
CL	22^05^	0.62
EtOH + CL	12^19^	7.61

## Data Availability

The data presented in this study are available within the article text, tables, and figures.
